# Interaction between *Pseudosuccinea columella* (Gastropoda: Lymnaeidae) and trematodes in a fasciolosis-endemic region in Espírito Santo, Brazil

**DOI:** 10.1590/S1984-29612025052

**Published:** 2025-10-17

**Authors:** Poliana Demuner Pereira, Jankerle Neves Boeloni, Hudson Alves Pinto, Isabella Vilhena Freire Martins

**Affiliations:** 1 Universidade Federal do Espírito Santo – UFES, Departamento de Medicina Veterinária, Laboratório de Parasitologia, Alegre, ES, Brasil; 2 Universidade Federal de Minas Gerais – UFMG, Departamento de Parasitologia, Laboratório de Biologia de Trematoda, Belo Horizonte, MG, Brasil

**Keywords:** Cercaria, Fasciola hepatica, trematodes, snails, xiphidiocercaria, Echinostoma, Cercária, Fasciola hepatica, trematódeos, moluscos, xifidiocercária, Echinostoma

## Abstract

This study aimed to identify larval forms of trematodes in *Pseudosuccinea columella* from rural properties with a history of bovine fasciolosis in the southern region of Espírito Santo, Brazil. A malacological survey was carried out on waterbodies from ten properties between June 2022 and March 2023. Aquatic snails were collected, identified, and evaluated for infection with trematode larvae by artificial photostimulation. The larvae found were morphologically characterized under a light microscope, and samples of the snails were subjected to histological analysis to observe larval trematodes in development in snails' tissues. A total of 678 specimens of *P. columella* were collected in nine properties, and larval trematodes were identified in 24 (4.08%) specimens from seven properties. A xiphidiocercaria morphotype, compatible with Haematoloechidae, was found in six properties. A morphotype of echinostome cercaria, compatible with the *Echinostoma* genus, was found in one property (2.17%). Larval *Fasciola hepatica* was not observed. Ecological studies are needed to investigate the environmental factors that may be related to the absence of natural infection of snails by *F. hepatica*, especially considering the presence of positive cattle in the evaluated properties. The data presented here reveal that *P. columella* a potencial vector role in the studied area.

## Resumo

Este estudo teve como objetivo identificar formas larvais de trematódeos em *Pseudosuccinea columella* de propriedades rurais com histórico de fasciolose bovina na região sul do Espírito Santo, Brasil. Entre junho de 2022 e março de 2023, foi realizado um levantamento malacológico em coleções aquáticas de dez propriedades. Moluscos aquáticos foram coletados, identificados e avaliados quanto à infecção por larvas de trematódeos por meio de fotoestimulação artificial. As larvas foram caracterizadas morfologicamente por microscopia de luz, e amostras de moluscos foram submetidas à análise histológica. Foram coletados 678 exemplares de *P. columella* em nove propriedades, e larvas de trematódeos foram identificadas em 24 (4,08%) exemplares de sete propriedades. Um morfotipo de xifidiocercária, compatível com Haematoloechidae, foi encontrado em seis propriedades, e uma cercária do tipo equinostoma, compatível com o gênero *Echinostoma,* em uma propriedade. Não foram observadas larvas de *Fasciola hepatica*. Estudos ecológicos são necessários para investigar os fatores ambientais que podem estar relacionados à ausência de infecção natural de caramujos por *F. hepatica,* especialmente considerando a presença de bovinos positivos nas propriedades avaliadas. Os dados aqui apresentados revelam que *P. columella* tem um potencial papel de vetor na área estudada.

## Introduction

*Fasciola hepatica* Linnaeus, 1758 is a digenetic trematode parasite of mammals with a global distribution (Mas-Coma et al., 2019). Fasciolosis primarily affects the liver of ruminants, such as cattle and sheep, causing economic losses, including reduced milk and meat production, low weight gain in infected animals, veterinary treatment costs, and liver condemnation during slaughter (Mas-Coma et al., 2019; Vieira et al., 2011). This species can also infect a variety of other animals including water buffalo, capybaras, and rodents. The zoonotic potential of *F. hepatica* should be emphasized, with cases reported in several countries worldwide (Mas-Coma et al., 1999). The epidemiology of fasciolosis is complex, involving the biology of gastropod intermediate hosts, particularly limnic snails of the Lymnaeidae family (Carneiro et al., 2013; El-Kouba et al., 2009; Martins et al., 2021).

Although fasciolosis is considered to be a disease that is spreading (Almeida et al., 2024; Vieira et al., 2011), few studies have focused on identifying the natural infection of snails of the Lymnaeidae family by *Fasciola hepatica* and other trematodes. Only a few studies have confirmed the occurrence of natural infection of lymnaeid snails with *F. hepatica* in the southern (Gonzales et al., 1974; Paraense, 1982) and southeastern regions of Brazil (Coelho & Lima, 2003; Lima et al., 2009; Oliveira et al., 2002; Ueta, 1980). Among the intermediate hosts of *F. hepatica* in Brazil, *Pseudosuccinea columella* (Say, 1817), previously known as *Lymnaea columella*, is the most widely-distributed species and is therefore present great epidemiological importance (Medeiros et al., 2014). The involvement of this species in the transmission of trematodes in wild animals, including species from the Haematoloechidae family and *Echinostoma* genus, has been reported in Brazil by several authors (Pinto & Melo, 2013a), but studies aiming to advance in the knowledge on these other non-*Fasciola* trematode transmitted by *P. columella* are still necessary in the country.

The identification of snail species in a given region, such as southern Espírito Santo, an area at risk of fasciolosis (Martins et al., 2012), is essential for understanding the dynamics of parasite transmission. Although there are records of natural infection of snails by *F. hepatica* in Brazil, such as the studies by Ueta (1980) and Amato et al. (1986), these remain limited to specific regions. Therefore, there is still a need for broader and more up-to-date research investigating the presence of trematode larvae in snails, especially in areas with a known risk of fasciolosis. Studies in this field can significantly contribute to understanding the disease's epidemiology and to the development of more effective and targeted control strategies.

Thus, this study aimed to assess the infection of *P. columella* by larval trematodes on rural farms located in an endemic area of bovine fasciolosis in southern Espírito Santo, Brazil.

## Materials and Methods

Analysis of farms with a history of fasciolosis was carried out by collecting data records from the Parasitology Laboratory at the Veterinary Hospital of the Federal University of Espírito Santo (HOVET-UFES) from 2020 to 2022. The experimental procedures were approved by the Ethics Committee on the Use of Animals (CEUA) of Alegre (UFES) under number 007/2021 at the Center for Agricultural and Engineering Sciences at the Universidade Federal do Espírito Santo, Brazil.

Based on a survey of farms with a history of fasciolosis, each property was visited to collect snails from bodies of water, such as rivers, lakes, animal water troughs, and all flooded areas. At least one visit was conducted on ten properties (Table 1), with additional visits determined based on accessibility. The duration of each malacological collection was from 30 min to 2 hours and the collection was conducted from June 2022 to March 2023.

**Table 1:** Number of specimens of *Pseudosuccinea columella*, number and percentage of infected snails, cercarian type and geolocation data from 10 farms with a history of bovine fasciolosis in southern Espírito Santo, Brazil, between June 2022 and March 2023.

**Farm number**	**Number of snails collected**	**Number of snails infected**	**% infected snails**	**Cercarian type**	**GPS**	**Municipality**
1	286	5	1.74	Xiphidiocercaria	20°45’57.8”S 41°27’18.2”W	Alegre
2	59	1	1.69	Xiphidiocercaria	-20.8030427,-41.4113547	Jerônimo Monteiro
3	107	1	0.93	Xiphidiocercaria	-20.782668, -41.406127	Jerônimo Monteiro
4	40	1	2.50	Xiphidiocercaria	-20.778218, -41.267498	Cachoeiro de Itapemirim
5	63	2	3.17	Xiphidiocercaria	-20.685353, -41.194105	Cachoeiro de Itapemirim
6	1	0	0.00	None	20°41’57.0”S41°34’35.1”W	Alegre
7	29	12	41.37	Xiphidiocercaria	-20.785736, -41.374082	Jerônimo Monteiro
8	92	2	2.17	Echinostome	20°47’15.8”S 41°24’42.0”W	Jerônimo Monteiro
9	0	0	0.00	None	20°47’58.2”S 41°24’17.0”W	Jerônimo Monteiro
10	1	0	0.00	None	20°43’35.9”S 41°23’18.1”W	Alegre
**TOTAL**	678	24	4.08			

Snails were collected by manual collection or captured with the help of a 30-cm diameter steel, round sieve and a plastic sieve with a nylon mesh when they were observed floating in the water. All specimens were collected and placed in a container with water from the environment for transport to the laboratory, where they were measured with calipers and identified morphologically (Brasil, 2008). The taxonomic identification of snails was confirmed by specialists and voucher specimens was deposited at the Malacological Collection of the Oswaldo Cruz Institute (CMIOC).

In the laboratory, snails were exposed to photostimulation every 10 days over a period of two months. Each exposure lasted four hours, by exposing the individuals to a light source from a 60-W incandescent bulb positioned 50 cm away. Each individual was placed in a well of a culture plate containing 10 mL of distilled water. Every 60 days, a new cycle of collection and light exposure was conducted, involving individuals from up to three different farms. After the 2 hours of exposure, the culture wells were examined using a stereomicroscope to analyze the emerging larval forms.

The larvae were collected directly from the multiwell culture plates, where the snails were individualized to stimulate the release of cercariae. The release was monitored with the aid of a stereomicroscope, allowing the visualization of the larvae moving in the liquid medium and their collection using a single-channel micropipette of variable volume (10–100 µL / 0.01–0.1 mL). The collected larvae were then transferred to slides and observed under a microscope. All larval forms collected, were placed on slides, stained with 2% Lugol’s solution, and examined under a light microscope (Brasil, 2008). Image records of emerging larval types were also taken with the aid of an Opticam Microscopy Technology 055R microscope (model LOP14003). The cercarial types were identified and classified based on the general biological characteristics of their respective groups, using the taxonomic keys described by Pinto & Melo (2013b).

Snails that were positive in the photostimulation test and 10% of those that did not show emerging larval forms were dissected using tweezers. The soft tissues were placed in Eppendorf tubes (2ml) containing 10% formalin solution for 24 hours. After fixation in 10% formalin, samples were processed following standard histological protocols, including dehydration, embedding in paraffin, and sectioning at 5 μm.

Humason’s (1979) histological processing method was used, which included staining with hematoxylin & eosin (HE), Masson's trichrome, and periodic acid–Schiff reagent (PAS). Different staining techniques were used to verify histological changes and specifically special stains (Masson's Trichrome and PAS) were performed with the aim of detecting histological changes such as fibrous connective tissue or mucus production by the digestive glands, respectively. The analysis focused on observing the sporocysts, redia, and cercaria in the tissues of the individuals, which were identified according to available literature (Brandolini & Amato, 2006; Pinto & Melo, 2013b; Souza et al., 1995; Sperandio, 2022). The images were captured using an Opticam Microscopy Technology 055R LOP14003 microscope. This study is essentially descriptive and exploratory, and were conducted descriptive statistical analyses.

## Results

From June 2022 to March 2023, ten farms were identified as positive for the presence of *F. hepatica*. During the study period, 947 limnic snails were collected, of which 678 (71.6%) were identified as *P. columella*. In addition, 213 specimens of *Biomphalaria glabrata* (Planorbidae), 13 *Melanoides tuberculata* (Thiaridae), and 43 *Stenophysa marmorata* (Physidae) were collected. Of the *P. columella* specimens, 24 (4.08%) tested positive in the photostimulation test (Table 1). Cercariae emerged predominantly during the first exposure, except at property eight, where emergence was recorded after 30 days. Two types of cercariae were identified: a xiphidiocercaria morphotype, found on six farms, and an echinostome cercaria morphotype, found only on farm eight, corresponding to 8.33% of the infected

### *P. columella* specimens

**Figure 1:**
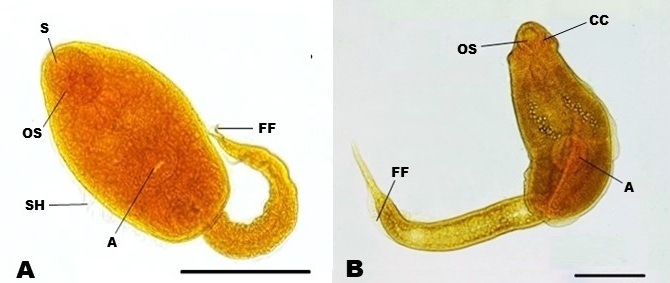
Cercarian types found in *Pseudosuccinea columella* collected in southern Espírito Santo, Brazil. A) Xiphidiocercaria - Ornata group compatible with species of the family Haematoloechidae. B) Larva of the type echinostome identified as *Echinostoma* sp.; scale bar: 100 µm. Abbreviations: (OS) oral sucker, (A) acetabulum, (S) stylet, (SH) sensory hairs, (FF) fin-folds, (CC) cephalic collar with spines.

The xiphidiocercaria morphotype (Figure 1A) presents the following general morphological characteristics: a well-developed oral sucker; well-developed stylet; body with sensory hairs; and tail, which is smaller than the body presenting fin-folds. This last characteristic classifies it as belonging to the Ornata group (Pinto & Melo, 2013b), which is produced by species of the family Haematoloechidae, which are pulmonary parasites of amphibians. These larvae were present in six of the ten farms analyzed. In total, 22 *P. columella* were found to harbor this morphotype, half of which (12) were collected from the same property (Farm 7).

The echinostome-type larvae (Figure 1B) were characterized by the presence of a cephalic collar with spines; a subterminal oral sucker; and a subequatorial acetabulum, which was slightly larger than the oral sucker. The tail was elongated and bore fin-folds. Based on the observed characteristics, these cercaria were associated with the genus *Echinostoma*. These cercaria were identified in two specimens of *P. columella* collected from Farm 8.

**Figure 2:**
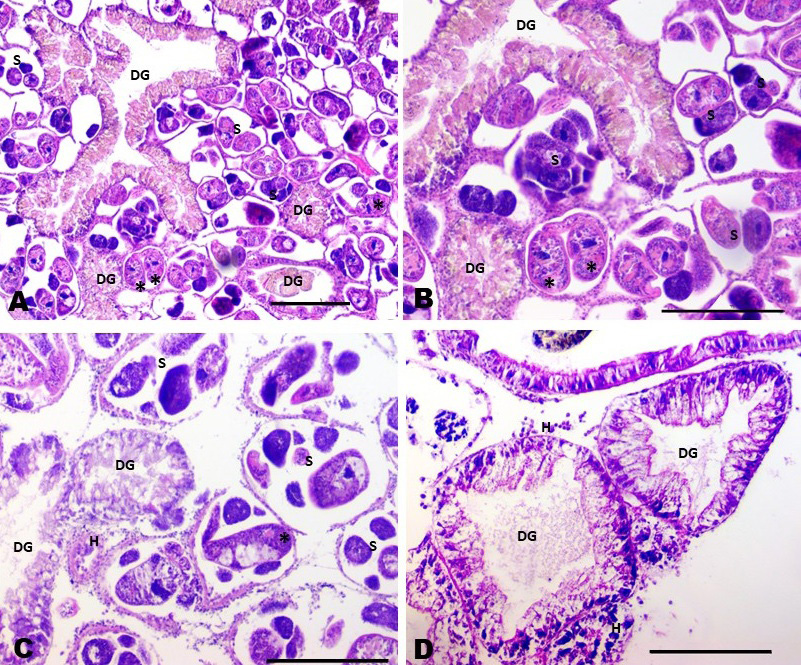
Photomicrography of the histopathology of *Pseudosuccinea columella* infected with larval stages of trematodes in southern Espírito Santo, Brazil. A) Connective tissue showing a high number of developing larvae near the digestive glands, stained with hematoxylin and eosin. B) Higher magnification of panel A. C) Connective tissue displaying larval forms and hemocytes near the digestive glands. D) Digestive glands without folds in their ducts. scale bar: 100 µm. (DG) digestive glands, (S) sporocyst, (*) structure with a body and tail similar to a cercaria, and (H) hemocyte (hematoxylin & eosin staining).

In the histopathological analysis, 91 specimens of *P. columella* were examined, including 24 individuals that presented a positive result in the artificial photostimulation test and 67 individuals (10%) that had a negative result. Of the latter, six (8.95%) presented with subclinical, identified histologically, with the same pattern observed in individuals positive for xiphidiocercaria. Histopathology indicated a high number of sporocysts with cercariae in development near the digestive glands, causing alterations, such as compression of the glandular ducts, reduction in epithelial height, and folds in the acini (Figure 2).

A large number of sporocysts were observed in the tissue, which caused folds in the acinar structure and narrowing of the lumen in the acinus. The presence of parasites in the connective tissue compressed the ducts of the digestive glands. The reduced height of the epithelium led to the formation of folds in the lumen of the ducts, which was not observed in the uninfected snails (Figure 2D). In the sporocysts, structures with bodies and tails resembling the larval form of cercaria were also observed. These structures appeared to be enclosed by a vesicle similar to that of sporocysts and were surrounded by rounded structures within the same vesicle (Figure 2C). Although the use of additional histological stains, such as Masson’s trichrome and PAS, was mentioned, these did not yield complementary results or were not effective for detecting alterations. Thus, Hematoxylin and Eosin (HE) was the predominant staining technique used to identify cellular alterations.

Although probable, it was not possible to link the trematode species found exclusively in the histopathological analysis with the xiphiocercariae found in the photostimulation tests. Structures suggestive of rediae were not visualized in the histopathological sections, indicating that they did not correspond to *F. hepatica* or Echinostomidae.

## Discussion

Understanding infection rates is crucial for evaluating the prevalence of these parasites in specific populations as well as for identifying potential risk factors associated with infection. Moreover, a detailed analysis of trematode larvae in snail tissues provides valuable data on the spatial distribution of infected hosts. In the current study, although the collections were made from farms positive for bovine fasciolosis and gymnocephalous cercariae, the morphotype of cercaria produced by members of the family Fasciolidae was not found in the 678 evaluated specimens of *P. columella*. This indicates that the natural infection of snails with *F. hepatica* in this area is low, which has been reported previously (Fernandez et al., 2018; Paviotti-Fischer et al., 2019).

This may be related to the life cycle of the parasite, environmental conditions, and the specific characteristics of snail populations. The presence or absence of cercariae may depend on factors such as snail population density, infection rates, competition between different snail species, and the period of collection (Galaktionov & Dobrovolskij, 2003).

The absence of *F. hepatica* cercariae in *P. columella* can be explained by several factors that go beyond the parasite's life cycle and common environmental conditions. Specific aspects of the local interaction between hosts, parasites and environment, such as water quality (pH, oxygenation), fluctuating temperature, rainfall regime and presence of aquatic vegetation, can create ecological barriers that hinder the development or transmission of the parasite (Alba et al., 2019). In addition, behavioral and reproductive characteristics of the *P. columella* population, such as feeding and breeding habits, can limit exposure or susceptibility to infection (Hadebe et al, 2025; Alba et al., 2019). Interspecific competition with other snails and the low density of intermediate hosts are additional factors that can reduce the natural prevalence of fascioliasis in the studied area (Ngcamphalala et al., 2022). These elements reinforce the complexity of the parasite dynamics and indicate that multiple ecological mechanisms must be considered to understand the distribution and intensity of infection in endemic environments.

A limitation of this study is its nine-month sampling window, which did not cover a full annual cycle; future research is needed spanning all seasons to assess seasonal peaks in infection.

Sampling may limit the detection of infections by trematodes; therefore, it is possible that the snails collected did not harbor *Fasciola* cercariae at the time of collection, resulting in the absence of these larvae in the tissues. Collections carried out in Piquete (SP) by Ueta (1980) identified the natural infection rates of *L. columella* by *F. hepatica* as 1.22% and 0.14% on different farms. These values, which are considered low, highlight the difficulty in detecting cercariae in naturally-infected snails, reinforcing the challenges of studying the transmission dynamics of the parasite under natural conditions. The distribution of trematode larvae in the intermediate snail host is supradispersed. Under natural conditions, we find few infected snails, but containing a high number of larvae, which constitutes an ecological strategy to avoid impact on the host population.

Before their death, a small number of cercariae were eliminated by the snails; however, most of these larvae were unable to complete their development and remained inside the rediae. In other individuals, natural infection with *F. hepatica* was confirmed only after the death of the snails, when numerous cercaria-containing species were observed (Ueta, 1980). Unlike the procedure adopted by Ueta (1980), the snails in this study were not crush environments, in order to preserve the integrity of the tissues for histopathological analysis. This decision allowed a more detailed characterization of the inflammatory changes associated with larval presence. On the other hand, the absence of crushing may have reduced the sensitivity in detecting larval stages that were not spontaneously released. To mitigate this limitation, a combination of photostimulation, stereomicroscopic observation, and histological analysis was used. Recognizing the gains in tissue detail, as well as the restrictions in detection, is essential for data interpretation and to guide future investigations.The discrepancies observed between the findings of photostimulation and the histopathological results can be attributed to methodological differences and the scope of each diagnostic technique. The main objective of photostimulation is to induce the release of viable cercariae, allowing their observation and morphological identification based on external characteristics. However, its effectiveness depends directly on the developmental stage of the larvae, the physiological state of the snail at the time of the test and the viability of the cercariae. Thus, immature, degraded or physiologically inhibited larvae may not emerge, resulting in false negatives.

On the other hand, histopathology allows a detailed evaluation of internal tissues, enabling the identification of larval structures at different stages of development, even when there is no emission of cercariae. This approach, however, also has limitations: histological examination is restricted to specific sections of the tissue, which may lead to the failure to visualize rediae or complete structures if these are absent in the slides analyzed or have been degraded during processing. Furthermore, in advanced infections, there may be lysis of the redial structures after the cercariae are released, making their detection in sections difficult.

Therefore, the two techniques are complementary: while photostimulation provides data on the active morphotypes and their release capacity, histopathology offers information on the presence, location and effect of the larvae on the host tissues. The discrepancies between the methods reinforce the importance of their joint application in order to increase diagnostic sensitivity and obtain a more comprehensive understanding of the infection.

In the present study, 4.08% of the specimens of *P. columella* were harbored xiphidiocercariae or echinostome cercariae. Cercariae are produced by trematodes that infect wild vertebrates. Other authors have reported these types of larval trematodes infecting lymnaeid snails in Brazil. A study conducted by Paviotti-Fischer et al. (2019) identified the presence of xiphidiocercariae larvae in 2/80 (2.5%) *P. columella* individuals, corresponding to an infection rate of 3.3% in the environment. Samples of snails collected by Oliveira et al. (2002) from the municipalities of Miracatu and Eldorado, located in the Vale do Ribeira region in the state of São Paulo, showed rates of 5.26% and 1.06%, respectively. This finding is consistent with the results of the current study, in which the infection rate in *P. columella* was 4.08%. The dynamics of cercarial emergence and the capacity of snails to support larval development can be influenced by various environmental factors and the physiological condition of the host. Understanding these factors is essential for developing effective strategies to monitor and control fascioloiasis, particularly in areas with high livestock populations.

The reported here in *P. columella* belong to the Ornata group Xiphidiocercaria morphology is compatible with that of other species in the family, Haematoloechidae, and similar larvae have been reported in Brazil (Carvalho et al., 2001). The life cycle involved aquatic insects (larval culicids) as secondary intermediate hosts and amphibians as definite hosts. The echinostome larvae reported herein may be morphologically associated with a species of the genus, *Echinostoma*. In Brazil, cercaria of *Echinostoma paraensei* Lie & Basch, 1967 were found in *P. columella* in the states of Rio de Janeiro and Minas Gerais (Lie & Basch, 1967; Maldonado et al., 2001; Pinto & Melo, 2013a).

Species of this genus require other snails or amphibians as second intermediate hosts, in which infective metacercariae occur; the definitive hosts are birds and mammals. Keiser & Utzinger (2005) reported that parasites of the genus, *Echinostoma,* can harm human health and affect food production in areas where infection is prevalent. There is also a risk of zoonosis with the potential transmission of parasites between animals and humans. *Pseudosuccinea columella* snails have been reported as naturally-infected with *Echinostoma caproni* in Egypt (Lotfy et al., 2017). To advance the specific identification of these two trematode species, further studies, including genetic characterization, are necessary.

The identification of cercariae as xiphidicercariae (Haematoloechidae) and of the *Echinostoma* type was based exclusively on morphology. Genetic characterization is essential to confirm taxonomic identity. Future studies should include molecular analyses to validate the identified species and expand knowledge about trematode diversity in the region.

Regarding histological analysis, Brandolini & Amato (2006) and Souza et al. (1995) described that infected snails exhibited a high number of sporocysts located in loose connective tissue. The larvae displayed hemocytic infiltration, resulting in the formation of a barrier resembling a granuloma, corroborating the findings of Sperandio (2022) and Tunholi et al. (2016). However, even in the digestive glands that did not contain larvae, hemocytic infiltration was observed, with an apparently higher intensity than that in the parasitized areas. This suggests that hemocytic infiltration may not be exclusively associated with the presence of larvae, but may be influenced by other factors or conditions present in the environment or within the digestive gland itself. In the current study, the high presence of larval forms in the connective tissue compressed the ducts of the digestive glands, causing epithelial-level alterations, as shown by Paviotti-Fischer et al. (2019).

The high number of sporocysts present in the digestive glands of *P. columella* documented in this study is consistent with the observations of Carvalho et al. (2001), who also noted that these sporocysts gave rise to xiphidiocercariae. However, Ueta (1980) mentioned the existence of infection with three different cercarian types in a single individual; however, the histological technique used did not elicit morphological differences between cercariae. Tunholi et al. (2016) showed that the presence of *E. paraensei* triggered a strong inflammatory reaction in the gonado–digestive gland complex of snail hosts. The current study indicated severe cellular disorganization and loss of organic function in digestive glands. In contrast, in uninfected snails, no larval stages were detected in the tissue sections of the digestive gland, which maintained normal integrity and function.

The presence of *Echinostoma* type cercariae and the investigation of *Fasciola hepatica* highlight the zoonotic relevance of the findings in this study. Both species are recognized human pathogens and may pose public health risks, especially in rural areas. Therefore, the results obtained may support epidemiological surveillance actions and prevention strategies aimed at both human and animal health.

Overall, the findings highlight the complexity of the interaction between *P. columella* and trematodes in an area endemic to bovine fascioliasis in Brazil. Studies that elucidate the biological aspects of these organisms in the region are needed to guide future strategies for controlling fasciolosis and may have a positive impact on the control of these diseases.

## Conclusion

The presence of *P. columella* in aquatic collections from most farms positive for bovine fasciolosis was confirmed. Although no specimens were found to be infected with *F. hepatica*, other larval trematodes (species of the families Haematoloechidae and *Echinostoma* sp.) were found to be cercariae. The absence of *F. hepatica* in the snails may be linked to local environmental factors, interspecific competition among snail species, or methodological limitations, such as the decision not to crush specimen, could have reduced detection sensitivity. Complementary, year-round ecological studies are therefore recommended to clarify how seasonality, water quality and snail population dynamics influence parasite transmission in this endemic area. The role of this snail in the transmission of other trematode species affecting wild animals in Espirito Santo State, Brazil, is reported here for the first time. The study highlights the challenge of understanding the environmental aspects related to parasite transmission, epidemiology, and control of fascioliasis in endemic areas, and the need for ecological studies to investigate factors such as interspecific competition, environmental barriers and methodological limitations that may influence infection dynamics and detection sensitivity.

In addition, these findings reinforce the importance of integrating public health and veterinary surveillance efforts, given the zoonotic potential of some of the identified trematodes.

## Data Availability

The raw data related to the collections and laboratory analyses carried out in this study are stored at the Laboratório de Parasitologia do Departamento de Medicina Veterinária da Universidade Federal do Espírito Santo, Alegre, ES, Brazil. These data include records of identification of trematode larvae in *Pseudosuccinea columella*, microscopic images, and histological results. The data can be obtained upon request to the corresponding author for purposes of verification and reproduction of the results.
